# Transforming Pain into Beauty: On Art, Healing, and Care for the Spirit

**DOI:** 10.1155/2014/789852

**Published:** 2014-04-16

**Authors:** Rachel Ettun, Michael Schultz, Gil Bar-Sela

**Affiliations:** Division of Oncology, Rambam Health Care Campus, 8 Ha'Aliyah Street, 35254 Haifa, Israel

## Abstract

From drawing to sculpture, poetry to journaling, and dance to music and song, the arts can have a major impact on patients' spiritual well-being and health. The arts empower patients to fulfill the basic human drive to create and give patients a sense of possibility. Through creative expression, patients regain a feeling of wholeness, individually and as part of the larger world. Although spiritual caregivers have made occasional use of the arts, it would be better for the arts to be seen as a pillar of spiritual care provision. This paper provides a model for arts-based spiritual care (chaplaincy) in oncology/hematology and elsewhere. We discuss how to match the art form intervention to the individual patient and give examples of many kinds of uniquely spiritual arts-based interventions. In life, there are occasional “caseuras,” or ruptures. Using a theoretical foundation drawn from theologian Michael Fishbane, our model of arts-based spiritual care bridges the experience of the caesura to a renewed sense of meaning, or spiritual reorientation, that can be discovered within the reality of illness. Additionally, the ambiguity and playfulness inherent to creative expression strengthen the patient's flexibility and resilience.

## 1. Introduction

The arts are an extremely valuable, if occasionally neglected, pillar of spiritual care (chaplaincy). A spiritual caregiver is an integral part of the interdisciplinary team in most hospitals [1, 2] and particularly in palliative care [3, 4], providing patients and family members undergoing a life crisis with an open heart and a listening ear. Through the spiritual caregiver's intervention, patients feel less alone, having shared their pain, even in the deepest sense. Patients also feel more connected to themselves and the world around them, having touched their personal world of the spirit and rediscovered their own spiritual resources. Spirituality is broadly defined: “spirituality is the aspect of humanity that refers to the way individuals seek and express meaning and purpose and the way they experience their connectedness to the moment, to self, to others, to nature, and to the significant or sacred” [4]. The arts refer to a wide range of means of creative expression, including visual arts with media such as paint, clay, stone, cloth and yarn, poetry or writing, music, dance and movement, storytelling, and drama. Enabling the patient to engage with the creative side of his being is part of the holistic approach to patient care as described by Sulmasy [5].

There are innumerable creative ways for a medical center to integrate the arts [6–11]. Lane has written extensively about current efforts to integrate the arts and health care [12–15]. Her “Arts in Medicine” program at Shands Hospital has hosted hundreds of artists, sculptors, dancers, musicians, and writers-in-residence to work with cancer, diabetes, and other patients, creating works of art at the patient's instruction or helping the patient become the artist [13]. Hundreds of hospitals and nursing homes around the world have adopted the concept of bringing artists into the health care setting. In any hospital, staff can play a crucial role in enabling artistic expression by asking patients what creative outlets they enjoyed in the past, either recent or remote, and bringing them the means to reengage creatively even while hospitalized, such as through music, drawing, movement, or journaling [12].

When these authors consider or study the ways in which these activities benefit the patient, the results are very often on the spiritual plane. Bailey summarizes five benefits of the arts for patients: (1) making the truths of life more real for us, (2) fulfilling a basic human need to be creative, (3) helping one to become a more whole person, (4) having the opportunity to experience joy, and (5) building bridges across diverse backgrounds [6]. Additionally, as Flannelly's study of spiritual needs found, we simply have a basic human need to appreciate art and beauty [16].

In Lane's study, patients described the process of their work as a transformative spiritual experience, going deep within themselves and by the end feeling greater love and compassion and a sense of connectedness to themselves, their bodies, and to what is beyond ourselves [14, 15].

Other spiritual benefits of the arts described are restoring a sense of identity, dignity, and community, as well as enabling patients' to express their own spiritual state [7], restoring a sense of meaning [10], making sense of one's experience and share that with others, enabling people to feel capable rather than limited, and enabling the heart to speak [17], and as an alternative means of communicating, especially when words are difficult [18, 19]. This last reason points to the additional significance of the arts in enabling a deeper form of communication with those suffering from cognitive decline and dementia [18].

Although the arts have other benefits for patients, their contribution to spiritual health and well-being is clearly strong. This indicates that it would be beneficial to integrate a professional spiritual caregiver into these arts programs to enable patients to direct their attention to the spiritual side of the creative experience. In that same vein, it would seem that all spiritual caregivers should be sure to incorporate some or all modalities for creative expression into their approach to providing spiritual care. Some of these articles describe doing just that [6, 7, 18]. Drawing on the theoretical approach of theologian Michael Fishbane, we will describe the model we have developed for the integration of the arts and spiritual care as well as expounding its benefits from a theological point of view.

## 2. Becoming a Creator

There is a deep connection between the spirit and the arts. The spiritual caregiver should make use of the arts as one of the most meaningful ways of connecting patients and all those around them to the healing power of the spirit within. The world of the arts is rich and broad, including the intangible relationship to aesthetics, music, drawing and sculpture, dance, architecture, poetry, and more. When man is created in the Bible, he is imbued to a certain extent with the nature of his Creator: “and God created man in His image; in the image of God He created him” (Genesis 1:27). In the following chapter, this act of creation is described differently: “the Lord God formed man from the dust of the earth. He breathed into his nostrils the soul of life, and man became a living soul” (Genesis 2:7, Judaica Press translation). The creative force has two aspects: the ability to create something from nothing and the ability to connect the material to the spirit, the ground with the soul.

Every patient, even the sickest or most disoriented, is a human being, created in the image of God and possessing inner spiritual resources that could aid in healing. We are referring to “healing” in the holistic sense, healing that is not only physical and that is possible even when no physical restoration is possible. Through the use of creative resources, the spiritual caregiver creates a language that connects the material with the spiritual and the brokenness of the body with the wholeness of the spirit. In this way, the patient connects to the divine creative quality within him. This experience of connection is itself one of healing, as distinct from curing [8].

The body is ailing, and the primary sense is one of injury and of what is lacking, a sense of being trapped. The spiritual caregiver, by inviting the creative act, opens the door of an alternative possibility [17], one that enacts that latent potential and fulfills our human need to create [6]. In place of the ever-growing feeling of meaningless waiting and powerlessness, there can be creation, vitality, and healing. Interestingly, in Hebrew, the words for creator and health share the same root. By entering into the creative process, a patient can switch from being the passive recipient of care to becoming active, choosing, and creating [17]. The act of choosing a shape and a color reinforces his basic freedom to choose life and spiritual growth until his final day. As Rabbi Kook, the first Ashkenazi chief rabbi of Israel, put it, “literature, drawing, and sculpture serve to bring to fruition all the spiritual conceptions submerged deep within the human soul” [20].

## 3. The Spiritual Impact of Hospitalization

A patient's experience is one of being moved, of being treated—he is not only unwell but also a passive object. Hopefully, this picture is just a temporary one, and the patient who, until recently, had been independent and active will shortly return to his normal functioning. Yet many patients become depressed during their hospital stay and leave the hospital feeling depleted, lacking in self-confidence and an inner sense of hope moving forward. Part of this feeling derives from the illness itself, from no longer seeing oneself as healthy, and from the worries that accompany this new reality. However, we would like to suggest that a significant portion of this difficult emotional or psychological state results from our caregiving attitude itself. Too often we fail to look at a patient as a whole person, instead relating to him or her through the lens of the disease, or the procedure he is undergoing—“the large intestine in room 7.” We need to strengthen the approach that looks to meet his spirit and his soul, and not only his body and his illness. We should remind ourselves that this frail person who has come for treatment also has a healthy, active core identity that defined him in the past and will hopefully continue doing so into the future.

## 4. Reassembling the Pieces into a New Whole

During illness and treatment, a patient is likely to feel like he has lost his sense of wholeness, as if he has been “disassembled.” The spiritual caregiver, by enabling a view of the fuller picture, invites the person trying to cope with his illness to place that sense of lost wholeness into a broader context. By entering the room and pulling out his paints, the glue, and the colored paper, the arts-inclined spiritual caregiver wordlessly reveals a new reality: there is a whole, undamaged picture. That wholeness is accessible to every person at every moment, regardless of what he may be going through. As Henri Matisse puts it, “the thought of a painter must not be considered as separate from his pictorial means, for the thought is worth no more than its expression by the means, which must be more complete… the deeper is his thought. I am unable to distinguish between the feeling I have about life and my way of translating it” [21]. Artists' lyrical descriptions of the feeling of merging with their creations are a source of inspiration and a reminder of the latent ability in all of us to move beyond the world of our health concerns and merge with that broader picture through our art. The Israeli poet and art critic Mordechai Goldman once said, “the creative endeavor enables an undifferentiated merging with one's subject, much like the connection between a nursing child and his mother's physical and mental state. The work is a kind of mirror, or kingdom of doppelgangers, in which its creator is reflected, just as the world of the mother is reflected in his childhood or as he is reflected in her world” [22].

In the hospital, we work with people whose movements have become rigid and limited. When a spiritual caregiver enters the room and offers the possibility of merging with the larger picture, even temporarily, he is suggesting movement and flow rather than pain and immobility. When that experience of merging ends, patient and spiritual caregiver often speak about it. This enables the patient to give a framework of meaning to the artistic endeavor. In this way, the creative act goes beyond just being a moment of forgetting and gains the imprimatur of being an experience of connection to the sublime. At the same time, we should be very careful not to overly put into words that which was expressed in a different language, the language of art.

In our spiritual care provision, we study and apply the unique approach put forth by the contemporary Jewish theologian Michael Fishbane. In considering the ruptures—the “caesura”—that suddenly interrupt a person's life and shake his very existence to the core, Fishbane suggests that the initial resource for coping with this rupture is the person's sensory world. The arts are the primeval language of the senses, even before words and language developed [23]. The arts are a middle ground between the caesura and a person's life's work. This space, in which a person develops this dual consciousness, is vital to the creation of a meaningful life and to one's spiritual development. By engaging his experience through art, a person experiences a reverberation of and glancing look at that dual consciousness as well as a means of maintaining it. As Fishbane writes, these ruptures may occur through events like illness: “these ruptures may occur in both nature and culture, through natural disasters and experiences of wonder, on the one hand, or via aesthetic achievements and poetic expressions, on the other. They rip the fabric of our own normal consciousness, bent on busyness and cultural buffers, and dispose us to a sense of unsettling finitude within vastness that exceeds all ordinary presumptions. Defenses fall and our fundamental fragility is suddenly manifest, at least for the moment. But if we hold firm, this crisis may yield a reformed consciousness or attitude” [23].

When we encounter a patient in the hospital who is in the midst of a caesura, the arts can provide an additional language. They are a bridge to the meaning of the reality with which he is contending. They are also a means of simultaneously holding the moment, as it is, with its attendant pain and limitations, and at the same time holding the eternal, the larger picture, and the sublime at which he “peeks through the cracks” (Song of Songs 2:9).

## 5. Putting the Theology into Practice

In 2007, the first author created Haverut (Friendship), an arts-based spiritual care nonprofit working in medical centers, including Hadassah, in Jerusalem, in memory of her daughter, Ruth, who passed away from cystic fibrosis at age 11. During her short life, Ruth drew heavily on the creative realm as a resource that gave her life and opened before her a world of infinite possibility even while she found herself facing a very limiting, terminal condition. Unable to get out of bed, receiving oxygen and a continuous flow of morphine, Ruth continued to draw, to write in her journal, to weave, and to listen to music. She was constantly in creative, vibrant contact with the spirit and with the soul. She ultimately chose the arts as her means of saying goodbye to her loved ones, drawing a small painting on canvas to give to each person, without a need for further words.  Box 1shares the story of a poem Ruth composed at age 6, after one of her lungs collapsed, together with the first author.

Elsewhere in the literature we find similar descriptions of the healing nature of the arts in spiritual care and the restoration of a sense of wholeness. Glenister quotes a patient's family member's description of the art room: “the act of creation was the process of healing and a celebration of everything broken” [7]. In Bras' words, “creation is an act of survival, growth, and development of the individual and the community” [9]. And Lane's patient interviews showed that patients engaging creatively went deep into themselves and came out feeling spiritually elevated [14, 15].

## 6. Matching the Art Form to the Patient

With such a wide variety of art forms available, how should the spiritual caregiver know what to suggest to which kinds of patients? The first step is to get a sense of the person through close listening and attunement. One thing to listen for is the language used by the patient—do they more often use words or images that are cognitive, visual, or auditory? That offers a preliminary means of indicating whether the appropriate intervention is a conversation or conceptual discussion (cognitive), visual arts, or music. Of course, the spiritual caregiver himself must feel comfortable with any particular art form before he can offer it to others. Lane suggests an alternative approach, asking the patient what kind of art form they liked in the past, and then trying to enable that for them but without necessarily providing additional guidance, while occasionally starting the process with guided imagery to enable the movement inward [15].

Certain forms of art are more widely accessible while others must be selected more carefully. Classic visual arts tend to be approachable for most people. Crafts that can be used, such as jewelry or a cloth journal, or even creating musical instruments, are even more accessible. Music (including singing), by contrast, because it makes noise and is less tangible, is more invasive and involves more of a kind of exposure, so it is primarily appropriate for patients who are already comfortable with it. Although one must be careful before introducing music, it can be a very powerful means of opening up the inner space. Songs that resonate in a patient's personal memory can be similarly strong. One approach to making music, singing, or art more accessible is to offer it to groups of patients. For example, many patients can work together on the same piece of art, as in the example in Box 2.

## 7. Not Art Therapy: The UniqueApproach of Arts-Based Spiritual Care

We have been exploring the spiritual benefits for patients of engaging in creative artistic activity. But we are not only suggesting a new way of looking at these kinds of services. There are also unique aspects of the practice of arts-based spiritual care, as distinct from art therapy, which we will now present.

The first element of our approach is the intention, or focus, of the spiritual caregiver. Just as a doctor and social worker at times both engage in conversation with patients but their focus is different, so too the art therapist and spiritual caregiver can both engage the patient's creative side while each bringing a very different intentionality to their work. The spiritual caregiver is focused on the divine image within the patient. Through their conversation and joint creative activity, the spiritual caregiver and the patient will explore together the patient's relationship with people and powers beyond themselves, their sources of meaning, or their way of understanding illness, God, and life.

The purpose, or product, of their artistic endeavor has a spiritual focus, albeit with “spiritual” conceived broadly. Sometimes that purpose has a strong connection to hope, prayer, or blessing, as with a patient who composes a personal prayer or poem, or a patient who creates an artistic rendering of a mantra—a helpful phrase or sentence to repeat—that will aid them throughout the day. Drawing a mandala provides the patient with a focal point for personal ritual practices such as meditation. Prayers and hopes can be expressed not only in words but also through objects, and together with the spiritual caregiver, patients might make prayer objects for their home, for their children, or for themselves, objects that facilitate connection to one's hopes and to the sacred in one's life.

In other instances, the goal of the artistic endeavor can be the creation of a personal ritual, especially rituals for transitions. This kind of ritual is a repeating set of practices that can help people throughout the time of the transition. One patient might design a ritual that will accompany them preparatory to a worrisome operation and in recovery, a ritual that could include a variety of art forms including song, text, and movement. Another patient could assemble a personal prayer book before starting chemotherapy, to be opened up on each treatment day, or other objects that they can regularly turn to or touch as sources of strength.

Another intersection of spirituality and art comes about through the holidays. Each holiday contains within it a particular set of meaning and resonances. By suggesting the patient make art connected to the holiday, the spiritual caregiver is creating an opening for a deeper engagement, in art and in conversation, with the patient's personal relationship with those issues raised by the holiday.

Perhaps an ideal approach is to combine engagement with both the left and the right brain. The spiritual caregiver might begin by sharing a text, such as a poem or a traditional religious text, in order to open up a discussion of the issues raised by the text that relate to the experience of illness. But by then shifting to a creative means of engaging with those issues, by involving the whole body and even the imagination, then we have now given the patient the freedom to translate the topic of the text to their own lives. The patient can now feel entirely free to add his own interpretation and to make the text, and its subject, his own. If it was a traditional text, then this engagement is in effect a part of becoming, oneself, part of the tradition, and that itself could be a powerful feeling. Enabling patients to engage with these issues through as many dimensions of their being as possible produces an experience that is more engaging, more validating, and more full of vitality.

## 8. Reversals and Surprises: Learning to Be Flexible

“You have transformed my lament into dancing; you undid my sackcloth and girded me with joy” (Psalms 30:12). The Psalmist attests to a reality in which reversals are possible. A person can go from a situation of mourning and lament to one of dancing and movement. He can succeed in leaving his suffering behind. The medieval philosopher, Maimonides, suggested that arts as a resource enables one to move away from a sense of depression and regret: “it is similar for one who was feeling the black bile [melancholy], and removed it via music and singing, strolling through the gardens and fine buildings, sitting among fine forms and the like, things that expand the soul and remove its gloomy thoughts. And the purpose of all this is to heal his body; and the purpose of healing his body—to obtain wisdom.” [24].

Fishbane further describes the potential for surprises: “we come to see that, in the deepest sense, no one time of day is like any other and that no one perspective can take it all in” [25].

The Hasidic master, Rabbi Nahman of Breslov, often expanded on the power of music and dancing to move someone out of a state of melancholy and help him heal his soul [26]. Regarding the power of music to heal and to unify the material and spiritual worlds, he wrote, “through melodies that contain the sound of crying, one can free the captives” [27] and, “for this is the essence of song—to gather in and clarify the good spirit” [28].

The creative act contains within it the essence of ambiguity and of accepting uncertainty. It is playful and full of surprises and new directions. Bringing the dimension of movement and cyclicality into what can be the linear world of medicine strengthens the individual's resilience for dealing with life's crises and changes by internalizing an enhanced sense of flexibility. In this process, the spiritual caregiver serves as a facilitator and witnesses to the movement the patient is undergoing as he reimagines the picture of his life situation. Box 2 illustrates this process of change and reinterpretation with another example from our work.

## 9. The Synergy of Art Interventions and Spiritual Care

There have been many qualitative discussions of the benefits of art interventions for a variety of patient populations and an increasing body of clinical studies. Music therapy has been found to reduce pain, improve mood, enhance communication, and reduce spiritual distress [10], and creative arts also help reduce pain [9]. For oncology or hematology patients, quantitative studies of art therapy interventions have shown a reduction in depression [29, 30], improved fatigue/sluggishness levels [29, 31], reduced anxiety [30], and improved coping resources [32], although these results have not always been consistently replicated [33, 34].

Spiritual care has been subjected to fewer clinical studies, but attending to patients' spirituality has been shown to have benefit in areas including enhanced quality of life [35, 36] and well-being [37] and reduced anxiety and despair [38, 39].

Our proposal, as we have modeled in our hospital-based work described here, is for spiritual caregivers to learn the arts and integrate them into their larger approach to care provision. Arts-based interventions, such as creative arts, music, and writing, ought to find their place beside traditional spiritual care approaches, such as conversation, prayer, the human connection, text study, exploring the world of meaning, breathing exercises, and guided imagery. Truthfully, all these are means of touching the human spirit. By bringing the arts into spiritual care, both are enhanced—spiritual care, as described in this paper, and the arts, by strengthening the elements of reflection, holding, and exploration in the spiritual dimension, all of which are enabled through the relationship with the spiritual caregiver.

## 10. Conclusion

The integration of arts into spiritual care offers a means of providing healing, at least for the spirit. The arts enable patients to reexperience wholeness, a sense of connectedness, and the joy of being a creator. Arts-based spiritual care provides an enhanced, multidimensional means of engaging with the spirit and of strengthening patients' resilience for the time ahead.

## Figures and Tables

**Box 1:**
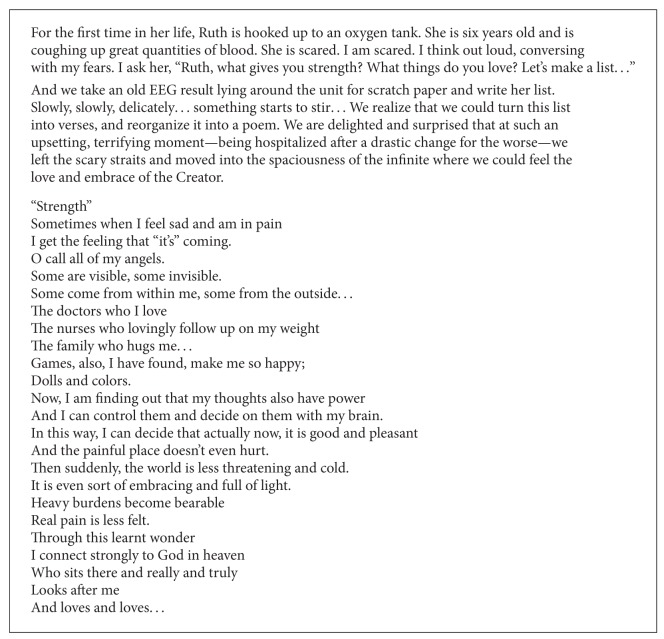
A 6-year old patient's poem—“Strength”.

**Box 2:**
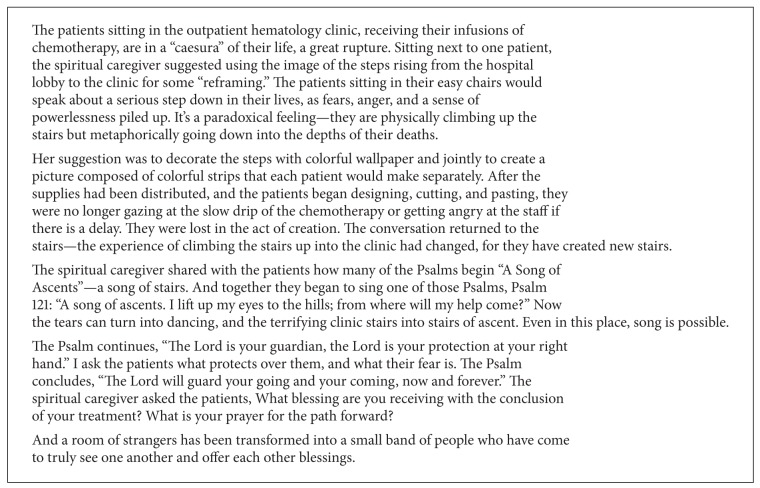
Reimagining the Stairwell.
